# Ductal ligation timing and neonatal outcomes: a 12-year bicentric comparison

**DOI:** 10.1007/s00431-021-04004-3

**Published:** 2021-03-13

**Authors:** Silvia Martini, Silvia Galletti, Wilf Kelsall, Emanuela Angeli, Marta Agulli, Gaetano Domenico Gargiulo, Si Emma Chen, Luigi Corvaglia, Yogen Singh

**Affiliations:** 1 Neonatal Intensive Care Unit, Department of Medical and Surgical Sciences, St. Orsola-Malpighi Hospital, University of Bologna, Via Massarenti 11, 40138 Bologna, Italy; 2 Neonatal Intensive Care Unit, Department of Paediatrics, Addenbrooke’s Hospital, Cambridge, UK; 3 Pediatric Cardiac Surgery Unit, St. Orsola-Malpighi Hospital, University of Bologna, Bologna, Italy; 4 Anaesthesiology and Intensive Care Unit, Cardio-Thoracic-Vascular Department, St. Orsola-Malpighi Hospital, University of Bologna, Bologna, Italy; 5 University of Cambridge School of Clinical Medicine, Cambridge, UK

**Keywords:** Patent ductus arteriosus, PDA ligation, Preterm infants, Bronchopulmonary dysplasia, Sepsis, Necrotizing enterocolitis

## Abstract

Patent ductus arteriosus (PDA) is common among extremely preterm infants. In selected cases, surgical PDA ligation may be required. The timing for PDA ligation may depend upon a variety of factors, with potential clinical implications. We aimed to investigate the impact of different surgical PDA managements on ligation timing and neonatal outcomes. Inborn infants < 32 weeks of gestation and < 1500 g admitted at two tertiary Neonatal Intensive Care Units that underwent PDA ligation between 2007 and 2018 were enrolled in this retrospective cohort study and split into the following groups based on their surgical management: on-site bedside PDA ligation (ONS) vs. referral to an off-site pediatric cardiac surgery (OFS). Neonatal characteristics, surgical timing, and clinical outcomes of the enrolled infants were compared between the groups. Multivariate analysis was performed to evaluate the impact of PDA ligation timing on significantly different outcomes. Seventy-eight neonates (ONS, *n* = 39; OFS, *n* = 39) were included. Infants in the ONS group underwent PDA ligation significantly earlier than those in the OFS group (median age 12 vs. 36 days, *p* < 0.001) with no increase in postoperative mortality and complications. The multivariate analysis revealed a significant association between PDA ligation timing, late-onset sepsis prevalence (OR 1.045, 0.032), and oxygen need at discharge (OR 1.037, *p* = 0.025).

*Conclusions:* Compared with off-site surgery, on-site bedside ligation allows an earlier surgical closure of PDA, with no apparent increase in mortality or complications. Earlier PDA ligation may contribute to reduced rates of late-onset sepsis and post-discharge home oxygen therapy, with possible cost-benefit implications.

**Table:** 

**What is known:** *• Ineffective or contraindicated pharmacological closure of a hemodynamically significant PDA may require a surgical ligation.* *• Available literature comparing the effect of early vs. late PDA ligation on the main neonatal morbidities has yield contrasting results*.	
**What is new:** *• The availability of a cardiac surgery service performing bedside PDA ligation allows an earlier intervention compared to patient referral to an off-site center, with no difference in postoperative mortality and complications compared to off-site surgery.* *• Earlier PDA ligation was associated with a lower prevalence of late-onset sepsis and of oxygen need at discharge, with possible cost-benefit implications.*

**Supplementary Information:**

The online version contains supplementary material available at 10.1007/s00431-021-04004-3.

## Introduction

Patent ductus arteriosus (PDA) is common among preterm neonates, with an estimated incidence of 60% in extremely-low-birth-weight infants [1]. The rate of spontaneous ductal closure is inversely related to gestational age, as the relative oxygen hyposensitivity of immature tissues and the scarceness of ductal medial muscles contribute to PDA maintenance [2]. The persistence of a significant systemic-to-pulmonary transductal shunt has been associated with adverse clinical outcomes, including pulmonary hemorrhage, bronchopulmonary dysplasia (BPD), intraventricular hemorrhage (IVH), necrotizing enterocolitis (NEC), and increased mortality [3–9].

If spontaneous closure is not achieved, PDA management includes supportive therapy and targeted pharmacologic treatment with cyclooxygenase inhibitors or paracetamol. However, in case of ineffective or contraindicated pharmacological closure of a hemodynamically significant PDA, surgical ligation may be required [10]. PDA ligation can be performed on-site, either at the infants’ bedside or in the operation room, or off-site, if a local or mobile pediatric cardiac surgery team is not available [11]. These different approaches may influence the timing for PDA ligation. Current literature comparing the effects of early vs. delayed ligation on neonatal morbidities highlights contrasting results [12–17], and the optimal surgical timing is still debated.

We aimed to assess whether different PDA ligation managements (on-site bedside ligation vs. referral to an off-site specialist pediatric cardiac surgical center) influence the timing of intervention and clinical outcomes in very preterm infants from two tertiary Neonatal Intensive Care Units (NICU) with different pediatric cardiac surgery capabilities.

## Materials and methods

Preterm infants < 32 weeks’ gestation born at the NICUs of Cambridge University Hospital (CUH, Cambridge, UK) and of St. Orsola-Malpighi Hospital (SOM, Bologna, Emilia-Romagna, Italy) between January 1, 2007, and December 31, 2018, were included in this retrospective study if, due to failed or contraindicated pharmacological therapy, they underwent surgical closure of PDA, judged hemodynamically significant (hsPDA) either on a clinical (hypotension, ventilator dependence, heart failure symptoms) and/or echocardiographic basis (left-atrial-to-aortic-root ratio > 1.5 and/or pulsatile left-to-right shunt and/or mean velocity in the left pulmonary artery > 0.6 m/s and/or evidence of diastolic reflow in the descending aorta, in coeliac or superior mesentery artery or in cerebral arteries). Major congenital malformations, including congenital heart defects (CHD), were an exclusion criterion.

Infants admitted to SOM NICU had their PDA ligated at bedside, with a timing of surgery dependent on the availability of surgeons and anesthesiologists, usually within 48–72 h from referral; however, in cases identified as urgent by the neonatologist or the pediatric cardiologist, the intervention was performed within 24 h. This team also provides bedside PDA ligations off-site, covering the whole NICU network of the Emilia-Romagna region, which includes other 7 tertiary NICUs.

In-house cardiac surgery was not available for CUH infants, as it ceased in December 2002 [18]; hence, they were referred to specialist pediatric cardio-thoracic centers in London, where PDA ligation was performed. In these cases, the ligation timing depended on the availability of the neonatal transfer team and of costs in the referral center.

Hence, based on their surgical PDA management, the infants included in this study were allocated in the following groups: on-site surgery (ONS, characterized by infants from SOM) vs. off-site surgery (OFS, characterized by infants from CUH).

The echocardiographic reports of the infants were retrospectively reviewed, and the following parameters from the latest pre-operative scan were recorded: PDA size and shunt characteristics, left-atrial-to-aortic-root ratio, evidence of reversed end-diastolic flow in the descending aorta, and/or anterior cerebral artery (when available). Prior pharmacological PDA management, including the related contraindications and adverse effects, was also reviewed; treatment failure was defined by PDA persistency after ≥ 2 pharmacological courses.

The following data were recorded from the study population: post-conceptional age, days of life and weight at surgical intervention, peri- and postoperative mortality, and complications (within 1 week from ligation or ascribable to surgery).

Clinical outcomes included mortality rates, IVH (grade 1 to 4) [19], periventricular leukomalacia (PVL) [19], NEC [19], bowel perforation [19], late-onset sepsis (defined as relevant symptoms with positive blood culture and/or C-reactive protein > 25 mg/L and > 5 days of antibiotic treatment after the first 72 h of life) [20], retinopathy of prematurity (ROP) [19], BPD (defined by the need for supplemental oxygen and/or positive-pressure respiratory support at 36 weeks’ post-conceptional age [PCA]) [19, 21], persistent pulmonary hypertension (PPHN, defined by the echocardiographic evidence of right ventricular hypertrophy or dilation with any among tricuspid regurgitation gradient > 40 mmHg or maximum velocity of the tricuspid regurgitant flow > 3 m/s, flat intraventricular septum or septal bowing into the left ventricle, right-to-left shunt across the PFO and/or the DA in the absence of CHD, and by the need for inhaled nitric oxide) [22, 23], length of hospital stay, time needed to achieve full enteral feeding (FEF), total duration of mechanical ventilation (MV), days off MV [24], and supplemental oxygen need at hospital discharge. NEC, IVH, and sepsis rates before PDA ligation were also noted. Infants deceased prior to 36 weeks’ PCA or before their first ophthalmological screening, FEF achievement, and hospital discharge were excluded from the evaluation of the related outcomes. Infants deceased prior to MV weaning were assigned a 0 value in the computation of MV-free days [24].

### Statistical analysis

Data distribution was verified using Shapiro-Wilk test. Continuous variables were expressed as median (interquartile range [IQR]). Clinical characteristics and outcomes were compared between the two groups using Chi-square or Fisher’s exact test for categorical variables and Mann-Whitney *U* test for continuous variables. Multivariate logistic regression models with backward elimination (likelihood ratio) including statistically significant neonatal variables at the univariate level and those variables with a known pathophysiological relationship were built to identify independent associations with the observed outcomes. Correlation matrix technique was used to evaluate multicollinearity among the model variables; absolute correlation coefficients > 0.5 indicated a high, not tolerable cross-correlation. The Hosmer-Lemeshow test was used to determine the goodness of fit of each logistic regression model. Statistical Package of Social Science (SPSS, IBM, Chicago, IL) software, version 26, was used for statistical analysis. Significance level was set at *p* < 0.05.

### Ethics

The study protocol was approved by the Ethic Committee “Area Vasta Emilia Centro–AVEC” (protocol no.231/2019/Oss) and by the clinical audit department at Cambridge University Hospitals (PRN no.800, study ID no.2000).

## Results

**Fig. 1:**
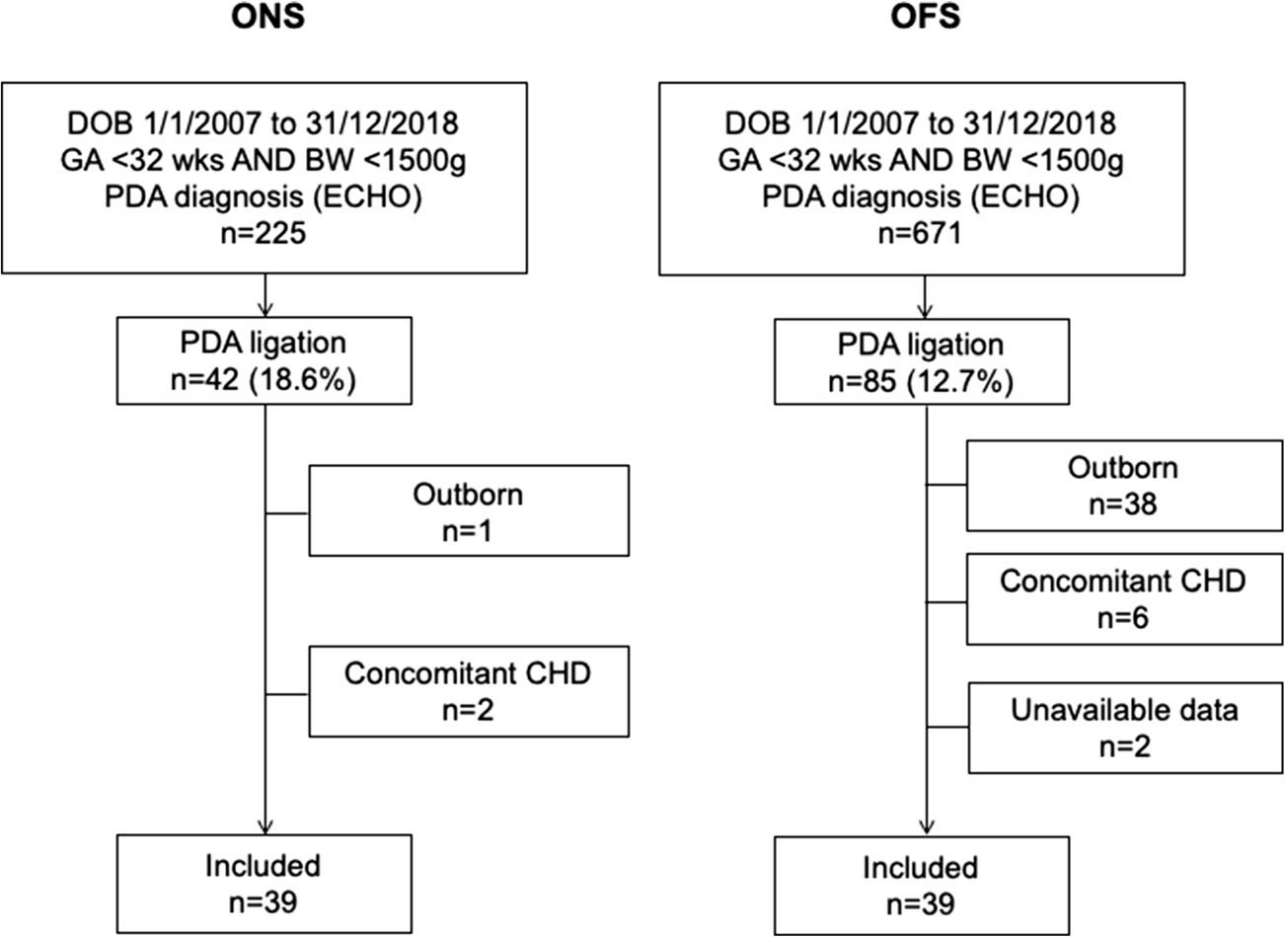
Flow chart of the study inclusion. CHD, congenital heart defect; DOB, date of birth; ECHO, echocardiography; ONS, on-site surgery; OFS, off-site surgery; PDA, patent ductus arteriosus

**Fig. 2:**
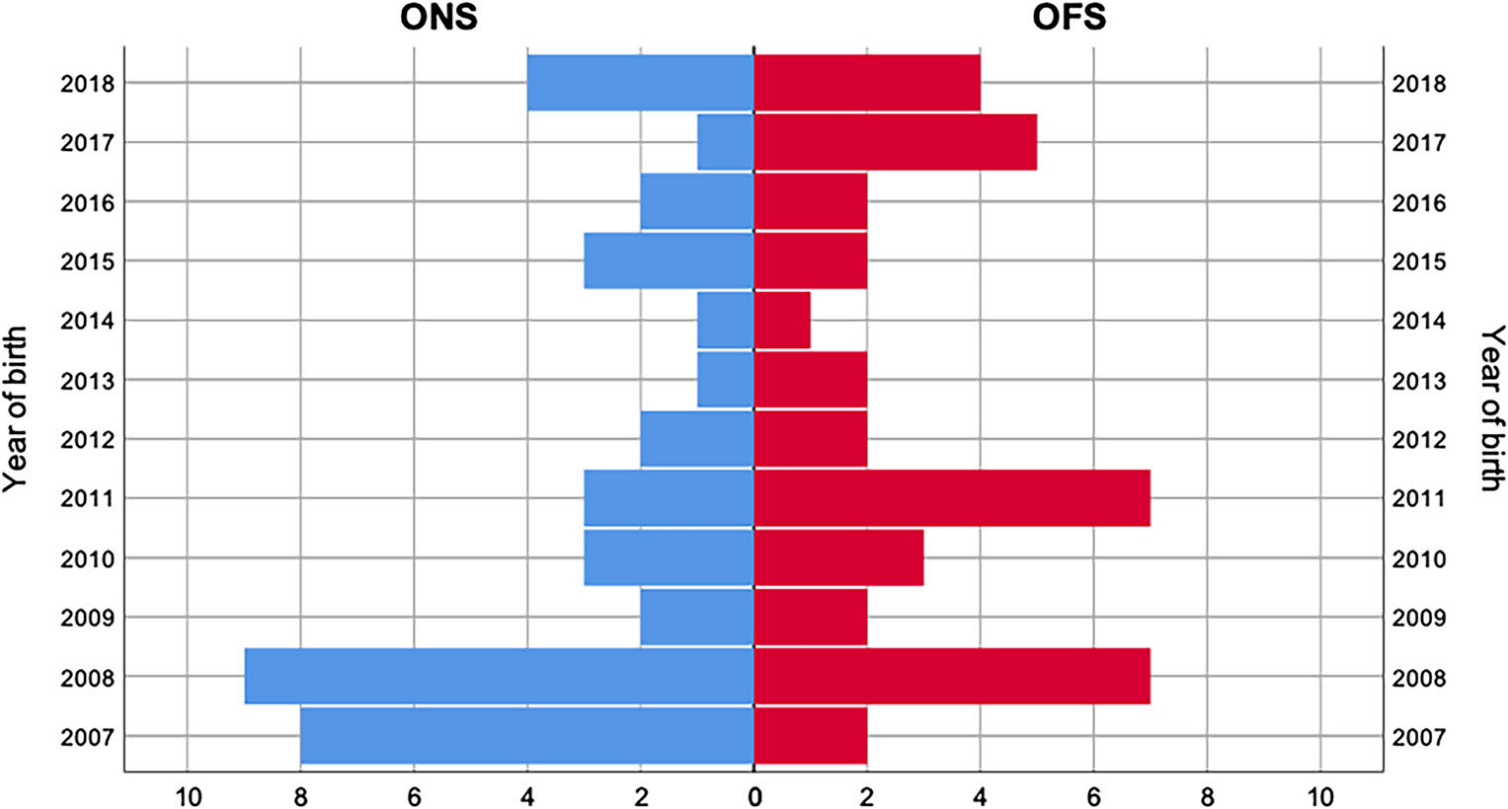
Yearly distribution of PDA ligation cases in the two study groups (on-site [ONS] vs. off-site surgery [OFS])

As illustrated in Fig. 1, 671 neonates from CUH and 225 from SOM were screened; of these, 47 were ruled out for the presence of exclusion criteria, whereas 2 CUH infants were excluded as data on their pharmacological PDA management were not available due to their participation to the Baby-OSCAR trial [25]. Seventy-eight neonates underwent PDA ligation and were included in the study: 39 in the ONS group and 39 in the OFS group. The yearly distribution of the study infants over the 12-year study period, illustrated in Fig. 2, did not differ significantly between the two centers.

Neonatal and PDA characteristics of the study groups are shown in Table 1. Infants in the ONS group showed slightly but significantly higher CRIB II scores and higher rates of C-section compared to those in the OFS group. No significant between-group differences were observed in the other neonatal variables that were evaluated. The first echocardiographic scan was performed at an earlier age in the ONS group compared to the OFS one. Nevertheless, pre-ligation echocardiographic features and pharmacological PDA management did not differ between the groups. In both centers, pharmacological closure was undertaken if the echocardiographic and clinical criteria for hsPDA described in the methods section persisted for > 48 h. Similar pharmacological dosages were used in the two groups (indomethacin, 0.2 mg/kg/day for up to 3 days; ibuprofen, 10 mg/kg on day 1, followed by 2 to 3 daily doses of 5 mg/kg; paracetamol, 15 mg/kg 6 h for 3 days). Since 2010, ibuprofen replaced indomethacin as first-line treatment. Paracetamol was introduced from 2016 and was mainly used in case of failure or contraindication of ibuprofen treatment. The number of pharmacological courses attempted before PDA ligation in each center and the medication used for each course are shown in Table 1; no significant differences were observed between the two cohorts. However, pharmacological PDA closure was attempted sooner in the ONS group compared to the OFS one (median age at first course: 3 [IQR 2–4] vs. 7 [IQR 4–10] days, *p* < 0.001).

**Table 1:** Clinical characteristics, echocardiographic features, and management of patent ductus arteriosus (PDA) in the on-site surgery (ONS) and off-site surgery (OFS) groups. Results of between-group comparisons are also reported; significant *p*-values are highlighted in italic

Neonatal characteristics	ONS (*n* = 39)	OFS (*n* = 39)	*p* Value
Gestational age (weeks), median (interquartile range [IQR])	25 (23.3–26.7)	25.1 (24.3–26.4)	0.350
Birth weight (g), median (IQR)	670 (560–820)	700 (600–797)	0.569
Apgar score at 5 min, median (IQR)	8 (5–8)	7 (6–8)	0.915
CRIB II score, median (IQR)	15 (12–16)	13 (11–14)	*0.016*
Sex (males), *n* (%)	24 (61.5)	24 (61.5)	1.000
Intrauterine growth restriction, *n* (%)	6 (15.4)	9 (23.1)	0.389
Delivery mode (C-section), *n* (%)	25 (64.1)	14 (35.9)	*0.013*
Antenatal steroids (complete course), *n* (%)	18 (46.2)	25 (64.1)	0.111
Age at first echocardiography (days), median (IQR)	2 (2–3)	4 (2–8)	*< 0.001*
Pre-ligation PDA characteristics			
Size (mm/kg), median (IQR)	4.1 (3–4.8)	4.2 (3.7–4.7)	0.245
LA:Ao ratio, median (IQR)	1.9 (1.7–2.1)	1.9 (1.8–2)	0.655
Pharmacological PDA closure attempted, *n* (%)	31 (79.5)	32 (82.1)	0.774
Number of pharmacological courses			
1, *n* (%)	14 (45.2)	14 (43.8)	0.611
2, *n* (%)	17 (54.8)	17 (53.1)	
3, *n* (%)	0 (0)	1 (3.1)	
Medication used for pharmacological PDA closure			
Ibuprofen, *n* (%)	33 (68.7)	29 (56.9)	0.154
Indomethacin, *n* (%)	8 (16.7)	17 (33.3)	
Paracetamol, *n* (%)	7 (14.6)	5 (9.8)	
PDA recurrence after pharmacological closure, *n* (%)	12 (30.8)	7 (17.9)	0.146
Age at PDA ligation (days), median (IQR)	12 (7–21)	36 (28–52)	*< 0.001*
Weight at PDA ligation (g), median (IQR)	630 (543–824)	1080 (868–1300)	*< 0.001*
Ongoing mechanical ventilation at PDA ligation, *n* (%)	34 (87.2)	23 (59)	*0.010*

IQR interquartile range, LA:Ao ratio left-atrium-to-aortic-root ratio, PDA patent ductus arteriosus

ONS infants underwent PDA ligation significantly earlier (median age 12 [IQR 7–21] vs. 36 [IQR 28–52] days, *p* < 0.001) compared to those that were transferred to a referral cardio-thoracic center. As expected, the weight at surgical intervention in the ONS group was significantly lower. Moreover, a significantly higher percentage of infants in the latter group required ventilation at the time of PDA ligation. Vital parameters at the end of surgery were available for the ONS group and have been provided as Supplementary Information. There was no difference in perioperative or postoperative mortality between the two groups. Post-surgical complications included pleural effusion (ONS, *n* = 1), vocal cord palsy (ONS, *n* = 1; OFS, *n* = 2), pneumothorax (OFS, *n* = 1), and transient left pulmonary artery narrowing (OFS, *n* = 1). As reported in Table 2, the rates of post-surgical complications and of sepsis onset within 1 week after surgery did not differ between the groups.

**Table 2:** Clinical outcomes in the on-site surgery (ONS) and off-site surgery (OFS) groups and results of between-group comparison. Significant *p*-values of between-group comparisons are highlighted in italic

Clinical outcomes		ONS (*n* = 39)	OFS (*n* = 39)	*p* Value
Surgical complications, *n* (%)		2 (5.1)	5 (12.8)	0.235
Post-ligation pulmonary hypertension, *n* (%)		3 (7.7)	1 (2.6)	0.615
Post-surgical sepsis (< 7 days after ligation), *n* (%)		2 (5.1)	4 (10.2)	0.675
Mortality, *n* (%)		8 (20.5)	3 (7.7)	0.192
Sepsis, *n* (%)		24 (61.5)	32 (82.1)	*0.044*
Sepsis, before PDA ligation, *n* (%)		8 (20.5)	26 (66.7)	*< 0.001*
Intraventricular hemorrhage	No IVH, *n* (%) Grade I–II, *n* (%) Grade ≥ III, *n* (%)	20 (51.3) 9 (23.1) 10 (25.6)	9 (23.1) 22 () 8 (20.5)	*0.007*
IVH, before PDA ligation, *n* (%)	No IVH, *n* (%) Grade I–II, *n* (%) Grade ≥ III, *n* (%)	20 (51.3) 9 (23.1) 10 (25.6)	12 (30.8) 19 (48.7) 8 (20.5)	0.055
Periventricular leukomalacia, *n* (%)		5 (12.8)	2 (5.1)	0.235
Necrotizing enterocolitis (Bell’s stage ≥ 2), *n* (%)		10 (25.6)	17 (43.6)	0.096
Necrotizing enterocolitis (Bell’s stage ≥ 2), before PDA ligation, *n* (%)		1 (2.6)	11 (28.2)	*0.003*
Time to full feeding (days), median (IQR)°		49 (33–61)	38 (24–51)	0.070
Mechanical ventilation (days), median (IQR)		29 (20–41)	33 (25–47)	0.185
Time off mechanical ventilation (days), median (IQR)		88 (44–98)	94 (74–110)	0.112
Bronchopulmonary dysplasia at 36 weeks, *n* (%)°		21 (70)	36 (94.4)	*0.004*
Retinopathy of prematurity°	No ROP Stage I, *n* (%) Stage II, *n* (%) Stage ≥ III, *n* (%)	11 (28.2) 7 (17.9) 9 (23.1) 7 (17.9)	14 (35.9) 7 (17.9) 10 (25.6) 8 (20.5)	0.987
Length of hospital stay (days), median (IQR)°		120 (90–138)	124 (119–143)	0.208
Oxygen need at discharge, *n* (%)°		6 (15.4)	19 (48.7)	*0.004*

°Infants deceased prior to outcome evaluation excluded from the analysis

*IQR* interquartile range, *IVH* intraventricular hemorrhage, *ROP* retinopathy of prematurity, *PDA* patent ductus arteriosus

The main study outcomes are detailed in Table 2. No between-group difference in the overall rates of mortality, PVL, NEC, time to FEF, and length of hospitalization was observed. The prevalence of IVH and sepsis during hospital stay was significantly higher in the OFS group compared to the ONS group (p = 0.007 and p = 0.042, respectively). When the pre-ligation period was analyzed separately, increased rates of sepsis (*p* = 0.007) and NEC (*p* = 0.003) were confirmed in the OFS group.

With regard to respiratory outcomes, MV duration and PPHN rates did not differ significantly between the groups; however, the prevalence of BPD at 36 weeks’ PCA and of oxygen need at discharge were significantly higher in the OFS group compared to ONS (*p* = 0.008 and *p* = 0.013, respectively).

Multiple logistic regression models were built to adjust the observed results for relevant clinical variables. Due to the evidence of a significant cross-correlation between the centers and PDA ligation timing (correlation coefficient = 0.725, *p* < 0.001), only the latter was included in the models. Each multivariate regression model also included CRIB II scores and the mode of delivery, which differed significantly between the study groups at the univariate analysis, and the year of birth, in order to address potential time effects due to the advances in neonatal care, occurred during the 12-year study period. Time to FEF was specifically added to the late-onset sepsis model, whereas MV duration and PPHN development were included in the models for BPD and oxygen need at discharge. No collinearity issues were observed. The Hosmer-Lemeshow test revealed an adequate fit for all the models (*p* > 0.05).

Results of the final regression models ensuing from backward selection are summarized in Table 3. Logistic regression confirmed a significant and independent correlation between PDA ligation timing, late-onset sepsis (*p* = 0.032, OR 1.045 [1.004–1.088]), and oxygen need at discharge (*p* = 0.025, OR 1.037 [1.004–1.170]). MV duration was significantly associated with both BPD (*p* = 0.016, OR 1.088 [1.016–1.165]) and oxygen need at discharge (*p* = 0.030, OR 1.041 [1.004–1.180]). A significant association between IVH development and CRIB II score (*p* = 0.015, OR 1.245 [1.043–1.486]), but not PDA ligation timing, was also observed.

**Table 3:** Results of the final logistic regression models (backward selection); significant *p*-values are highlighted in italic

Model	*R*^2^	Variables	B	SE	OR (95% CI)	*p* Value
Late-onset sepsis	0.268	CRIB II score	0.183	0.110	1.201 (0.968–1.491)	0.096
		Time to FEF	0.028	0.015	1.028 (0.999–1.959)	0.061
		Ligation timing	0.044	0.020	1.045 (1.004–1.088)	*0.032*
		Constant	-3.758	1.712	0.023	0.027
Bronchopulmonary dysplasia	0.437	CRIB II score	0.239	0.160	1.269 (0.927-1.738)	0.137
		MV duration	0.084	0.035	1.088 (1.016–1.165)	*0.016*
		Ligation timing	0.040	0.025	1.041 (0.992–1.092)	0.106
		Constant	-4.698	2.365	0.009	0.047
O_2_ need at discharge	0.328	MV duration	0.040	0.019	1.041 (1.004–1.180)	*0.030*
		Ligation timing	0.036	0.016	1.037 (1.004–1.170)	*0.025*
		Constant	-3.119	0.853	0.044	< 0.001
Intraventricular hemorrhage	0.145	CRIB II score	0.219	0.090	1.245 (1.043–1.486)	*0.015*
		Ligation timing	0.025	0.015	1.026 (0.996–1.056)	0.094
		Constant	-3.088	1.397	0.046	0.027

*FEF* full enteral feeding, *MV* mechanical ventilation, *OR* odds ratio, *SE*, standard error

## Discussion

The present results show that the availability of a local cardiac surgery service performing bedside ligation allows an earlier surgical closure of PDA compared to patient referral to an off-site tertiary cardio-thoracic center, with no difference in postoperative mortality and complication rates. We also identified a significant association between a later off-site PDA ligation, an increased prevalence of late-onset sepsis, and increased oxygen requirements at discharge.

Surgical ligation is considered when pharmacological PDA closure is either contraindicated or has failed to elicit clinical and echocardiographic improvement, with persistent mechanical ventilation dependence [10]. Based on the local availability of pediatric cardio-thoracic surgical services, operative practices are prone to certain inter-institutional variability. When these services are not available, either locally or as a mobile team provided from a referral center, the infants’ transfer to a referral center for PDA ligation is common practice; however, off-site transportation often requires reasonably stable clinical condition and may be associated with adverse events including hypothermia, line dislodgement, and unplanned extubation [10].

In the present study, bedside PDA ligation more than halved the surgical timing compared to off-site interventions, with similar rates of postoperative complications, confirming the safety of this procedure, when performed by an experienced team.

The timing of PDA ligation in the referral center also depends on cost availability, which may contribute to some delay in surgical intervention, prolonging the persistence of an hsPDA. On the other hand, bedside PDA ligation has proved to be a safe and effective technique, with surgical outcomes comparable to operating room closure, low rates of postoperative infection, and reduced resource utilization [26, 27].

Nevertheless, additional factors, such as the earlier age of the ONS cohort at the first echocardiographic evaluation and at the first pharmacological attempt of PDA closure, may have partially contributed to the significant differences in PDA ligation timing observed between the two study groups.

Current literature comparing neonatal outcomes in relation to the surgical timing of PDA ligation is limited. In 2015, Ibrahim et al. [16] reported a shorter MV duration and a lower fraction of inspired oxygen at 24 h postoperatively in preterm infants who underwent PDA ligation within the first 3 weeks, compared to a later intervention; however, they found no between-group difference in BPD development. Two years later, Youn et al. [17] retrospectively investigated neonatal outcomes after early (< 2 weeks) and late (≥ 2 weeks) PDA ligation: No difference in the main morbidities and mortality occurred, but a significant association between PPHN development during the first week of life and late ligation was observed.

In the present study, the prevalence of BPD was higher in both groups compared to that reported in extremely preterm infants by other cross-continent neonatal network studies [28–30]. While the correlation with BPD did not reach statistical significance in the multivariate model, we found a significant independent association between PDA ligation timing and oxygen need at discharge. Since no difference in the length of NICU stay was observed, it is likely that infants requiring post-discharge oxygen supplementation represented the subgroup with most severe BPD.

The association between BPD development and PDA treatment is an active matter of debate: Current evidence has not clarified whether this morbidity may be related to the negative effects of the ductal shunt itself, or indirectly to its treatment modality. Increased BPD rates in preterm infants undergoing PDA ligation compared to pharmacological treatment have been reported [31–33] and confirmed by a meta-analysis [34]; however, data from a large American network showed that reduced ligation rates were not accompanied by a consistent BPD decrease [35]. Schena et al. [36] observed that surgically treated neonates who developed BPD underwent PDA ligation significantly later compared to those without BPD. The prolonged exposure of the developing lung to pulmonary overflow has been proposed as a likely underlying mechanism, although other variables could have contributed to this finding. The presence of methodological biases, such as the lack of standardized definitions for both BPD and hsPDA [37] and the adoption of different approaches for PDA closure, may have also contributed to these controversial results [10].

The cerebral hemodynamic disturbances caused by an hsPDA may be involved in the development of IVH, especially during postnatal transition. We observed a higher IVH prevalence in OFS infants, who underwent PDA ligation significantly later, but logistic regression showed no association between the ligation timing and IVH development. Moreover, all the cases in the ONS group occurred before surgical ligation, consistent with the typical early timing of IVH onset [5, 38]. On the other hand, independent association between IVH occurrence and increasing CRIB II scores was observed, consistently with available literature [39].

As for gastrointestinal complications, multiple NEC cases occurred during the pre-ligation period in infants undergoing a later PDA surgery, whereas only one infant in the ONS group developed NEC before ligation. The detrimental effects of a persistent hsPDA on splanchnic circulation, which may increase the risk for ischemic-related gut complications, support this observation [40, 41]. However, other variables, such as different feeding managements adopted by the two centers in the presence of an hsPDA, may have contributed to this finding. Of note, most NEC cases in the OFS group occurred after FEF achievement.

We observed a significant correlation between the prevalence of sepsis and PDA ligation timing, independent of other covariates. A major risk factor for late-onset sepsis in NICU settings is the presence of central venous catheters (CVC) [42]. The higher pre-ligation NEC prevalence observed in the OFS group may have protracted the permanence of CVC for therapeutic and nutritional reasons. Moreover, PDA ligation itself requires a perioperative withholding of enteral feeds, further prolonging the need for long lines while building up enteral nutrition in the post-surgical phase, thus further increasing the risk of sepsis. However, since data on CVC permanence were not available for part of the study cohort, this hypothesis cannot be confirmed by the present data.

The following study limitations need to be acknowledged. The relatively small size of the study sample prevented the use of more refined and accurate statistical strategies (e.g., the use of a propensity score), whereas the retrospective patient inclusion could not exclude a possible selection bias. Moreover, the bicentric nature of the study and its 12-year period might have contributed to the heterogeneity of the study sample. To address this issue, the observed results were adjusted also for the year of birth. However, given the small percentage of neonates requiring PDA ligation, which is estimated around 6% of infants with hsPDA [1], multicenter studies will be necessary to achieve powered comparisons of different surgical approaches. Eventually, data on ventilation modalities were not fully available for the whole cohort and therefore were not included in the study outcomes.

Although our results need to be further validated, there appear to be cost-benefit implications. The clinical complications that were found to be associated with later PDA ligation have a major impact on health care services. These costs may not offset the financial resources required to set up a local service of pediatric cardiac surgery, with a mobile operative team providing bedside ligation, which is currently unavailable in the UK.

## Conclusion

The availability of a local cardiac surgery service performing bedside PDA ligation allows an earlier surgical timing compared to patient referral to an off-site tertiary cardiac surgery center, with no increase in postoperative mortality and complications. Although larger studies are needed to confirm this finding on a global scale, decreasing the timing for PDA ligation may also aid to reduce the occurrence of specific clinical associations (such as decreased need of home oxygen therapy on discharge and reduced rate of late-onset sepsis), with potential implications on health care resources.

## Supplementary Information

ESM 1 (DOCX 17 kb)

## Data Availability

Data are available from the corresponding author upon reasonable request.

## Abbreviations

BPD: Bronchopulmonary dysplasia
CUH: Cambridge University Hospital
CVC: Central venous catheter
hsPDA: Hemodynamically significant patent ductus arteriosus
FEF: Full enteral feeding
IVH: Intraventricular hemorrhage
MV: Mechanical ventilation
NEC: Necrotizing enterocolitis
NICU: Neonatal Intensive Care Unit
OFS: Off-site surgery
ONS: On-site surgery
PCA: Post-conceptional age
PDA: Patent ductus arteriosus
PPHN: Persistent pulmonary hypertension of the newborn
PVL: Periventricular leukomalacia
ROP: Retinopathy of prematurity
SOM: St. Orsola-Malpighi Hospital

## Code Availability

N/A.

## References

[1] Ngo S, Profit J, Gould JB, Lee HC (2017). Trends in patent ductus arteriosus diagnosis and management for very low birth weight infants. Pediatrics.

[2] Lago P, Bettiol T, Salvadori S, Pitassi I, Vianello A, Chiandetti L, Saia OS (2002). Safety and efficacy of ibuprofen versus indomethacin in preterm infants treated for patent ductus arteriosus: a randomised controlled trial. Eur J Pediatr.

[3] Kluckow M, Evans N (2000). Ductal shunting, high pulmonary blood flow, and pulmonary hemorrhage. J Pediatr.

[4] Dollberg S, Lusky A, Reichman B (2005). Patent ductus arteriosus, indomethacin and necrotizing enterocolitis in very low birth weight infants: a population-based study. J Pediatr Gastroenterol Nutr.

[5] Evans N, Kluckow M (1996). Early ductal shunting and intraventricular haemorrhage in ventilated preterm infants. Arch Dis Child Fetal Neonatal Ed.

[6] Clyman RI (2013). The role of patent ductus arteriosus and its treatments in the development of bronchopulmonary dysplasia. Semin Perinatol.

[7] Havranek T, Rahimi M, Hall H, Armbrecht E (2015). Feeding preterm neonates with patent ductus arteriosus (PDA): intestinal blood flow characteristics and clinical outcomes. J Matern Neonatal Med.

[8] Ognean ML, Boantă O, Kovacs S, Zgârcea C, Dumitra R, Olariu E, Andreicuţ D (2016). Persistent ductus arteriosus in critically ill preterm infants. J Crit Care Med.

[9] Rozé J-C, Cambonie G, Marchand-Martin L, Gournay V, Durrmeyer X, Durox M, Storme L, Porcher R, Ancel PY, Hemodynamic EPIPAGE 2 Study Group (2015). Association between early screening for patent ductus arteriosus and in-hospital mortality among extremely preterm infants. JAMA.

[10] Weisz DE, Giesinger RE (2018). Surgical management of a patent ductus arteriosus: is this still an option?. Semin Fetal Neonatal Med.

[11] Gould DS, Montenegro LM, Gaynor JW, Lacy SP, Ittenbach R, Stephens P, Steven JM, Spray TL, Nicolson SC (2003). A comparison of on-site and off-site patent ductus arteriosus ligation in premature infants. Pediatrics.

[12] Jaillard S, Larrue B, Rakza T, Magnenant E, Warembourg H, Storme L (2006). Consequences of delayed surgical closure of patent ductus arteriosus in very premature infants. Ann Thorac Surg.

[13] Teixeira LS, Shivananda SP, Stephens D, van Arsdell G, McNamara PJ (2008). Postoperative cardiorespiratory instability following ligation of the preterm ductus arteriosus is related to early need for intervention. J Perinatol.

[14] Sung SI, Choi SY, Park JH, Lee MS, Yoo HS, Ahn SY, Chang YS, Park WS (2014). The timing of surgical ligation for patent ductus arteriosus is associated with neonatal morbidity in extremely preterm infants born at 23-25 weeks of gestation. J Korean Med Sci.

[15] Fonseca E, Georgiev SG, Gorenflo M, Loukanov TS (2014). Patent ductus arteriosus in preterm infants: benefits of early surgical closure. Asian Cardiovasc Thorac Ann.

[16] Ibrahim MH, Azab A, Kamal NM (2015). Outcomes of early ligation of patent ductus arteriosus in preterms, multicenter experience. Med (United States).

[17] Youn Y, Moon C-J, Lee J-Y, Lee C, Sung IK (2017). Timing of surgical ligation and morbidities in very low birth weight infants. Medicine (Baltimore).

[18] Sivakumar S, Lee L, Tillett A, Wells F, Dunning J, Kelsall AW (2007). Outcome of ligation of the persistently patent arterial duct in neonates as performed by an outreach surgical team. Cardiol Young.

[19] (2018) Vermont Oxford Network. Manual of Operations, Part 2, Release 22.1

[20] Cailes B, Kortsalioudaki C, Buttery J, Pattnayak S, Greenough A, Matthes J, Bedford Russell A, Kennea N, Heath PT, neonIN network (2018). Epidemiology of UK neonatal infections: the neonIN infection surveillance network. Arch Dis Child Fetal Neonatal Ed.

[21] Isayama T, Lee SK, Yang J, Lee D, Daspal S, Dunn M, Shah PS, for the Canadian Neonatal Network and Canadian Neonatal Follow-Up Network Investigators (2017). Revisiting the definition of bronchopulmonary dysplasia: effect of changing panoply of respiratory support for preterm neonates. JAMA Pediatr.

[22] Fuloria M, Aschner JL (2017). Persistent pulmonary hypertension of the newborn. Semin Fetal Neonatal Med.

[23] de Boode WP, Singh Y, Molnar Z (2018). Application of neonatologist performed echocardiography in the assessment and management of persistent pulmonary hypertension of the newborn. Pediatr Res.

[24] Contentin L, Ehrmann S, Giraudeau B (2014). Heterogeneity in the definition of mechanical ventilation duration and ventilator-free days. Am J Respir Crit Care Med.

[25] Outcome after Selective early Closure of patent ductus ARteriosus (PDA) in extreme preterm infants: a randomised controlled trial (Baby-OSCAR). http://www.isrctn.com/ISRCTN84264977. Accessed 24 Jan 2019

[26] Avsar MK, Demir T, Celiksular C, Zeybek C (2016). Bedside PDA ligation in premature infants less than 28 weeks and 1000 grams. J Cardiothorac Surg.

[27] Metin K, Maltepe F, Kır M, Bilen Ç, Sökmen A, Oto Ö, Uğurlu B (2012). Ligation of patent ductus arteriosus in low birth weight premature infants: timing for intervention and effectiveness of bed-side surgery. J Cardiothorac Surg.

[28] Stoll BJ, Hansen NI, Bell EF, Shankaran S, Laptook AR, Walsh MC, Hale EC, Newman NS, Schibler K, Carlo WA, Kennedy KA, Poindexter BB, Finer NN, Ehrenkranz RA, Duara S, Sanchez PJ, O'Shea TM, Goldberg RN, van Meurs KP, Faix RG, Phelps DL, Frantz ID, Watterberg KL, Saha S, Das A, Higgins RD, for the Eunice Kennedy Shriver National Institute of Child Health and Human Development Neonatal Research Network (2010). Neonatal outcomes of extremely preterm infants from the NICHD neonatal research network. Pediatrics.

[29] EXPRESS Group (2010). Incidence of and risk factors for neonatal morbidity after active perinatal care: extremely preterm infants study in Sweden (EXPRESS). Acta Paediatr.

[30] Ancel P-Y, Goffinet F, Kuhn P (2015). Survival and morbidity of preterm children born at 22 through 34 weeks’ gestation in France in 2011. JAMA Pediatr.

[31] Kabra NS, Schmidt B, Roberts RS (2007). Neurosensory impairment after surgical closure of patent ductus arteriosus in extremely low birth weight infants: results from the Trial of Indomethacin Prophylaxis in Preterms. J Pediatr.

[32] Mirea L, Sankaran K, Seshia M (2012). Treatment of patent ductus arteriosus and neonatal mortality/morbidities: adjustment for treatment selection bias. J Pediatr.

[33] Weisz DE, Mirea L, Rosenberg E, Jang M, Ly L, Church PT, Kelly E, Kim SJ, Jain A, McNamara PJ, Shah PS (2017). Association of patent ductus arteriosus ligation with death or neurodevelopmental impairment among extremely preterm infants. JAMA Pediatr.

[34] Weisz DE, More K, McNamara PJ, Shah PS (2014). PDA ligation and health outcomes: a meta-analysis. Pediatrics.

[35] Hagadorn JI, Brownell EA, Trzaski JM, Johnson KR, Lainwala S, Campbell BT, Herbst KW (2016). Trends and variation in management and outcomes of very low-birth-weight infants with patent ductus arteriosus. Pediatr Res.

[36] Schena F, Francescato G, Cappelleri A, Picciolli I, Mayer A, Mosca F, Fumagalli M (2015). Association between hemodynamically significant patent ductus arteriosus and bronchopulmonary dysplasia. J Pediatr.

[37] Gomez Pomar E, Concina VA, Samide A, Westgate PM, Bada HS (2018). Bronchopulmonary dysplasia: comparison between the two most used diagnostic criteria. Front Pediatr.

[38] Levene MI, Fawer CL, Lamont RF (1982). Risk factors in the development of intraventricular haemorrhage in the preterm neonate. Arch Dis Child.

[39] Lee SM, Lee MH, Chang YS (2019). The clinical risk index for babies II for prediction of time-dependent mortality and short-term morbidities in very low birth weight infants. Neonatology.

[40] Clyman RI (2006). Mechanisms regulating the ductus arteriosus. Biol Neonate.

[41] Martini S, Aceti A, Galletti S et al (2020) To feed or not to feed: a critical overview of enteral feeding management and gastrointestinal complications in preterm neonates with a patent ductus arteriosus. Nutrients 12 10.3390/nu12010083 PMC7019993 31892190

[42] Cantey JB, Milstone AM (2015). Bloodstream infections: epidemiology and resistance. Clin Perinatol.

