# Supplementary material for: Ductal ligation timing and neonatal outcomes: a 12-year bicentric comparison
Source: Eur J Pediatr. 2021 Mar 13;180(7):2261–70. doi: 10.1007/s00431-021-04004-3 (PMC7955694; doi:10.1007/s00431-021-04004-3)
Supplement: Supplementary file 1 — (DOCX 17 kb) [file 431_2021_4004_MOESM1_ESM.docx]

**Ductal ligation timing and neonatal outcomes: a 12-year bicentric comparison.**

*European Journal of Pediatrics*

Silvia Martini^*^, Silvia Galletti, Wilf Kelsall, Emanuela Angeli, Marta Agulli, Gaetano Domenico Gargiulo, Si Emma Chen, Luigi Corvaglia and Yogen Singh

**Corresponding author*: Silvia Martini, MD - Neonatology and Neonatal Intensive Care Unit

St.Orsola-Malpighi Hospital, Via Massarenti 11 – 40138 Bologna, Italy. Phone/Fax number (39) 051 342754; e-mail: silvia.martini9@unibo.it

**Supplementary Information.** Vital parameters at the end of on-site surgery (ONS).

| **Parameter** | **ONS (n=39)** |
| --- | --- |
| *Body temperature (°C), median (IQR)* | 36.2 (35.9-36.5) |
| *Mean arterial blood pressure (mmHg), median (IQR)* | 36 (31-44) |
| *pH, median (IQR)* | 7.31 (7.27-7.35) |
| *Base excess (mmol/l), median (IQR)* | 1.3 (-3.2; 4.9) |
| *PaO_2_/FiO_2_ ratio* | 1.23 (0.88-1.49) |
